# Maternal education and sibling inequalities in child nutritional status in Ethiopia

**DOI:** 10.1016/j.ssmph.2022.101041

**Published:** 2022-02-11

**Authors:** Hilde Bras, Jornt Mandemakers

**Affiliations:** a University of Groningen, Department of History, Oude Kijk in 't Jatstraat 26, 9712 EK, Groningen, the Netherlands; b Atlas Research, Amsterdam, the Netherlands

**Keywords:** Child nutritional status, Maternal education, Gender, Birth order, Ethiopia, Sub-Saharan Africa

## Abstract

In many societies child nutritional status varies between siblings because of parental gender and birth order preferences and differential intra-household resource allocation. While more educated women have been found to improve children's nutrition overall, it is unclear whether they also buffer sibling inequalities in nutritional status. We study the interplay between parental preferences, maternal education, and sibling inequalities in child nutritional status in Ethiopia, the second most populous country in sub-Saharan Africa, with high rates of malnutrition, rapid socio-economic change, urban fertility decline, and low, but increasing female education. We base our analysis on a pooled sample of the 2011/12, 2013/14, and 2015/16 waves of the Ethiopian Socioeconomic Survey using 8275 observations from 4402 children between the age of six months and 9 years old nested in 1687 households. Results from multilevel and fixed effects models show sizable gender and birth order differences in nutritional status. Boys had a better nutritional status than girls and earlier born children had a better nutritional status than later born children, both in terms of height-for-age and weight-for-age. More educated mothers buffered sibling inequalities in nutritional status according to birth order, but not according to gender. The height penalty of being a higher order child disappeared for children whose mothers had about eight years of education or more (primary school finished/some secondary school). The beneficial impact of maternal education, counteracting some within-family inequalities, asks for continued investments in girls' and women's education.

## Highlights

• The maternal education and siblings' nutrition relationship is not well understood.
• We found steep gender and birth order gradients in child nutritional status.
• Educated mothers buffer sibling inequalities in nutrition according to birth order.
• Educated mothers do not compensate inequalities between boys and girls.
• Maternal education's influence is independent of household wealth and residence.

## 1 Introduction

Women's education is often hailed as key to improved child nutrition in low and middle income countries (Smith et al., 2003; UNICEF, 2011). Specifically, development programs hinging on gender equality and public policies promoting gender equity as a means to ensure economic growth drive efforts to increase school attendance and educational attainment of girls and women (Nussbaum, 2000, 2011; World Bank, 2012). First, educated women have been found to wield a positive influence on child health and nutrition because they have more knowledge of, and greater access to information on child feeding and care practices, hygiene, and sanitation (Glewwe, 1999; Semba et al., 2008; Thomas et al., 1991). Second, higher-educated women are more likely to have better-paid jobs in the formal sector and thus more resources to provide children with nutritious food and health care (Brauner-Otto et al., 2019; Nankinga et al., 2019). Third, education has been found to improve women's bargaining position in the household, enabling them to allocate more and/or better food and health care to children (Kunto & Bras, 2018; Lépine & Strobl, 2013; Sen, 2001).

Although the focus on maternal education has contributed to better explanations of child nutrition, few studies have attempted to understand how mother's education may differentially impact a child's nutritional status according to his or her position among siblings. In many cultures, children from the same family fare very differently in life (Steelman et al., 2002). Their sibling position, i.e. the child's gender and birth order amid their siblings, is an important determinant of their health outcomes, including their nutritional status (Behrman, 1988; Collin, 2006; Garenne, 2003; Horton, 1988; Jayachandran & Pande, 2017). Such sibling inequalities may result from differential intra-household food allocation, discrepancies in informal care-giving practices, and access to formal health care. Ultimately, they are related to the varying roles, norms, and preferences regarding siblings in different family systems, religions, and social groups, as well as to the impact of modernization (Rossi & Rouanet, 2015). While mother's education has been found to improve child nutrition overall, little is known about whether more educated women compensate the health position of the worst-off children in the household. A difficulty in previous research addressing this question is that gender and birth order inequalities are not always measured by comparing brothers and sisters within the same household (e.g. Bose, 2011). A number of recent studies have, however, shown that studying siblings sheds more light on how inequalities come into existence, and whether parents buffer these differences, or not (Fledderjohann et al., 2014; Hadley et al., 2008; Jayachandran & Pande, 2017; Kunto & Bras, 2018) Hence, one of the key contributions of our study is a within-family approach, which controls for alternative explanations that may explain gender and birth order inequalities.

We examine the intersection of maternal education, child preferences, and child nutritional status in Ethiopia, a low-income, drought-prone country in East Africa with the second largest population of the African continent. Although Ethiopia is one of the fastest growing economies in the region, it remains one of the poorest, with a per capita income of $790 (World Bank, 2020). The prevalence of stunting and acute malnutrition has decreased over the past decade, but remains high, with 38% of children under 5 years stunted and 10% wasted (USAID, 2018). Undernourishment has decreased from 52% of the population in 2000 to 21% in 2016 (FAO, 2018). Ethiopian women's average educational level is relatively low, but in the cities, mostly in Addis Ababa, female educational levels are rising (Barro & Lee, 2013; UNDP, 2018). To what extent do more educated Ethiopian mothers moderate sibling inequalities in nutritional status?

## 2 Background

In many societies, health inputs, such as food, informal care practices, and access to health care, are not allocated equally across all children in the family. Intra-household resource allocation may vary according to children's gender and birth order based on for instance parental discriminatory preferences and budget constraints (Conley, 2005; Den Hartog, 1973; Mock et al., 1994; Pongou, 2013).

First, gender preferences may differentiate the health and well-being of brothers and sisters. Girls may receive less nutrition relative to their brothers because of labour market returns, which favour men in most settings (Rosenzweig & Schultz, 1982) or because of social norms and preferences of particular social, religious, and ethnic groups (Dyson & Moore, 1983; Madjdian & Bras, 2016). However, male gender biases also exist. Some studies in Asia and sub-Saharan Africa have found that boys are more stunted than girls (Garenne, 2003; Madjdian et al., 2018; Svefors et al., 2020; Wamani et al., 2007).

Previous research on Ethiopia has observed a female child gender bias in intra-household resource allocation and nutritional status (Collin, 2006; Fafchamps et al., 2009; Hadley et al., 2008; Koohi-Kamali, 2008). Koohi-Kamali (2008), for instance, presented evidence of discrimination against girls based on the consumption patterns of households' adult members. A study by Hadley et al. (2008) examining gender bias in food insecurity experiences of Ethiopian adolescents found that girls were more likely than boys to report being food insecure, although no differences in their households’ food insecurity status existed. Gender differences were largest in severely food insecure households. The same pattern was also observed when male-female sibling pairs living in the same household were compared (Hadley et al., 2008).

Such gender disparities are in line with the sociocultural context of Ethiopia where patriarchy is the dominant cultural model (Poluha, 2004), associated with patrilineal and patrilocal property ownership and inheritance practices (Dodoo & Frost, 2008; Goody, 1976). In everyday life, adolescent Ethiopian boys generally enjoy more rights and privileges than girls and are often able to spend more time outside their homes allowing them to seek food elsewhere (Hadley et al., 2008; Poluha, 2004). Boys are also more often supported and receive small amounts of money to help them feed during the day, while girls are less likely to experience the same privileges (Mains, 2007).

A second driver of sibling inequalities in nutrition are preferences regarding birth order. Eldest (son) preference, primogeniture, and special roles allotted to the eldest, or for that matter the youngest, are examples of this (Conley, 2005; Den Hartog, 1973; Mock et al., 1994). Cultural norms and practices and expected economic returns of specific children may drive such disparities. Children of lower parity, particularly the first-born, are often predetermined to inherit the land, or have more chances to be gainfully employed on the labour market. If an important motive for having children is old age security, then the eldest children, who become economically independent first, may also be more favoured (Horton, 1988).

Birth order inequalities may also be related to budget constraints or resource dilution, pertaining to the amount of resources available for a given child in a particular phase of the household cycle (Downey, 2001; Steelman et al., 2002). In general, children of higher parity have to share the household's resources with a larger number of siblings when they are young than those who were born earlier in the sibling row. The larger the family, the greater the dilution of resources and therefore the lower the nutritional status of the child (Downey, 2001; Hatton & Martin, 2010). Finally, the availability of food for different generations of siblings may diverge as a result of for instance wars, disasters, famines, or new food technologies.

Previous evidence from Ethiopia suggests that birth order is related to children's nutritional status. Kebede (2005), using the Ethiopian Rural Household Survey, found that stunting increased with birth order. Similarly, a study by Collin (2006) revealed that there was significant sibling inequality in nutritional status with higher parity children being more heavily stunted than their lower birth order siblings.

There is broad empirical support for the positive association between maternal education and child nutrition (Cunningham et al., 2015; Haddad, 1999; Lépine & Strobl, 2013), although some authors have suggested that the relationship with health outcomes may not be causal and that maternal education may act as a proxy for the socioeconomic status of the family and/or the geographic area of residence (Bras & Smits, 2021; Desai & Alva, 1998). In line with this, several studies in Ethiopia have observed a positive relation between children's nutritional status and mother's educational level (Abdulahi et al., 2017; Alemayehu et al., 2015; Brhane & Regassa, 2014; Christiaensen & Alderman, 2004; Fafchamps et al., 2009; Gurmu & Etana, 2013; Kimhi, 2004). For instance, Gurmu and Etana (2013) observed that children of more educated mothers had a better nutritional status compared to children of less educated mothers. Kimhi (2004) found that the economic position of women had a positive effect on child nutritional status in southern Ethiopia. A study that investigated the effect of women's bargaining power on intra-household allocation of welfare in rural Ethiopia found that female empowerment benefitted child nutrition (Fafchamps et al., 2009).

However, few studies in sub-Saharan Africa, let alone in Ethiopia, have examined whether maternal education moderates the relationship between parental gender and birth order preferences and child nutritional status. A study by Collin (2006) observed that Ethiopian parents compensated their higher birth order children, who were more heavily stunted, at the expense of lower birth order children, but the study did not address differences by maternal education and buffering between sons and daughters. Following previous findings from studies in Western settings (Grätz & Torche, 2016; Hsin, 2012), we expect that more educated Ethiopian mothers buffer gender inequalities (H1) and birth order inequalities (H2) in nutritional status among siblings.

## 3 Data and Methods

### 3.1 Data

We base our analyses on a pooled sample of the first 2011/12, second 2013/14 and the third 2015/16 waves of the Ethiopian Socioeconomic Survey (ESS). The ESS is a large-scale nationally representative longitudinal household panel dataset collected by the Central Statistics Agency of Ethiopia in collaboration with the World Bank (CSA 2018). The first wave was limited to rural regions and small towns.

In the ESS, the nutritional status of children (height and weight) was assessed for children up to five in the first, up to seven in the second, and up to nine years old in the third wave. We selected the 3441 households (out of 5462 households) that had children of at least six months old to age nine (7473 children). Information on nutritional status was available for at least one child in 3160 households (6258 children, 11,992 observations). After removing outliers for nutritional status according to WHO guidelines for measuring child growth (WHO, 2006) the number of observations was reduced to 6187 children (3141 households, 11,787 observations). We further excluded 944 observations of children with an unknown or deceased mother at the time of observation. Finally, because we are mainly interested in within-family differences, we excluded families with only one observed child (1332 mothers/children were excluded). The final sample comprised 8275 observations of 4402 children nested in 1687 households, 1697 mothers (some households comprised multiple mothers due to polygynous marriages/extended families; we treat these as separate families). The sample differed slightly by outcome because of differing numbers of excluded outliers (7657 observations for height-for-age and 8228 for weight-for-age). There were 184 mothers with four or more observed children in the final sample for height-for-age and 203 for weight-for-age. The maximum was six for both outcomes. We used listwise deletion of missing values, except for a number of control variables where we included missing/unknown categories in order to maximize the number of observations.

### 3.2 Measures

In each wave of the ESS, children's nutritional status was assessed. Weight was measured in kilograms, rounded to 1 decimal. Height was measured in cm, also rounded to 1 decimal. The child's height was measured lying down if the child was younger than two years of age, otherwise standing up. As children had not attained their final stature and growth curves differ by sex, we standardized their stature by sex and age using the WHO child growth reference standards (WHO, 2006). We derived two standardized measures: height-for-age (stunting) and weight-for-age (underweight). Outliers were excluded based on WHO guidelines (absolute Z-scores larger than 5 or 6 on the WHO references scores).

Key independent variables are gender (boy/girl), birth order (1–7+), and maternal education. Birth order was capped at 7 or higher. Mother's educational level was measured as the highest educational qualification in years.

We included a number of control variables at the child and mother level. At the child level, we controlled for child age as factors such as changing family dynamics and accessing school feeding programs, which cannot be directly measured, may correlate with age. Moreover, different risks of gendered mortality selection may possibly impact gender gaps in nutrition at earlier vs. later stages of childhood. We also controlled for wave of measurement (1–3), as general circumstances faced by children in Ethiopia may have changed over the course of the study, which could have driven birth order differences in child nutrition. For instance, between 2011 and 2012, a severe drought affected East Africa causing a grave food crisis (OCHA 2011).

We included a wide range of variables to capture between-family differences that may indicate resources available to children, which possibly correlate also with maternal education. To control for possible resource dilution effects related to sibship size, we controlled for the number of siblings of a child (time-varying); we capped this variable at 7+ to reduce the influence of outliers. The family's socio-economic status was measured by using a wealth index (time-varying) of a selection of nine essential and luxury items (whether the family has a blanket, matrass/bed, watch/clock, (mobile) phone, radio, tv, stove, form of transportation, jewels). We also controlled for the biological father's highest attained educational level (in years), which could vary between children of the same mother due to remarriages. And we included a time-varying indicator of whether the biological father was deceased. We included a number of additional controls that may be related to family size and child rearing practices, these were mother's age at first birth (<18, 18–19, 20–24, 25–29, 35+, missing), mother's religion (Ethiopian Orthodox, Protestant, Muslim, other/missing), and the mother's marital status (married, polygynous marriage, never married, divorced/separated, widowed, missing) (time-varying). We further controlled for region in Ethiopia to take the large regional variation in Ethiopia into account. Finally, we included a variable charting the urbanization status of the community of residence (rural, small town, or big city), to control for community-level effects driving the association between maternal education and child nutrition.

### 3.3 Analytical strategy

The data were analyzed with multi-level and fixed effects models. We first ran the multi-level models to examine child and family differences in nutritional status for height-for-age and weight-for-age. Variance was partitioned into three levels: observations (level 1), which were nested within children (level 2), which were nested within mothers/families (level 3). Note that some families had multiple mothers due to polygynous marriages, but we decided not to include an additional fourth household level as there were relatively few such families. We begin with this baseline model (model 1, Table 3) that includes child characteristics, the number of siblings, the mother's educational level, and a host of control variables to get an overall impression of the educational gradient in childhood nutritional status in Ethiopia.

Next, we present fixed effects models that control for all observed and unobserved family-specific characteristics (models 2–4, Table 3). These models show the best estimates of the effects of birth order and gender on nutritional status given that controlling for a specific set of observable variables is always limited. We thus focus on within-family (between sibling) differences in nutritional status as estimated by the fixed effect models. We first estimated a mother/family fixed effect model (model 2), thereby controlling for all time-constant family background factors. In model 3 we included interactions of birth order and gender with maternal education to test our hypotheses. In a further fixed effect model (model 4) we included additional interactions with the main family controls (of model 1) to examine whether the mother's educational interactions could be ‘explained away’ by the socioeconomic status of the household or geographic residence and to see whether these factors affect within-family differences as well. Note that the standard errors were adjusted to correct for using multiple observations of children (1.9 observation per child on average).

## 4 Results

### 4.1 Descriptive results

Descriptive statistics are depicted in Table 1. The mean height-for-age and weight-for-age were well below zero in this sample, indicating that this is an undernourished population. There were slightly more observations of boys than girls (51.6%). There is a good representation of children of different birth orders; about a third were first/second born, about a third fifth or later born, and the remaining third were in the middle. The mean birth order was 3.5. Children were on average 3.9 years old (1413 days). Each consecutive wave contributes more observations, probably because the second and third wave expanded the age criteria for inclusion. Children had on average 3.2 siblings. Fathers were better educated than mothers, with 3.4 years on average compared to 2.1 years. The children's households had on average three of the selected essential and luxury items that comprised the wealth index. The mothers were mostly Christian (34% Ethiopian Orthodox, 24% Protestant) and 40% was Muslim. Mothers started childbearing early; almost 50% of the children had a mother who had her first child before turning 20. Most children lived with married parents, although there were sizeable groups whose mothers were in a polygynous marriage, or whose mothers divorced/separated or were widowed. The large majority of observations were from rural areas.

**Table 1:** Descriptives (unstandardized) (*N* = 8275).

	Mean	S.d.	Range
*Child characteristics:*			
Height-for-age (z-score) (*N* = 7657)	-1.45	1.92	-6–5.89
Height (cm.) (*N* = 7657)	94.3	16.60	56–161
			
Weight-for-age (z-score) (*N* = 8228)	-1.24	1.39	-5.98 - 4.9
Weight (kg.) (*N* = 8228)	13.6	4.38	3.4–38.3
			
Gender (Girl = ref.) (*i*)	.484		0–1
Boy	.516		0–1
Birth order (1 = ref.) (*i*)	.153		0–1
2	.201		0–1
3	.188		0–1
4	.170		0–1
5	.127		0–1
6	.085		0–1
7+	.076		0–1
Birth order (*i*)	3.476	1.80	1–7
Child age (in days) (*i*) (*t*)	1413	780	180–3299
Wave (wave 1 = ref.) (*t*)	.225		0–1
Wave 2	.348		0–1
Wave 3	.427		0–1
Number of siblings (*t*)	3.220	1.86	0–7
Father's education (*i*)	3.381	4.01	0–17
Father deceased (*i*) (*t*)	.093		0–1
*Household characteristics*			
Mother's education	2.129	3.49	0–17
Wealth (*t*)	3.125	1.91	0–9
Mother's religion (Orthodox = ref.)	.341		0–1
Protestant	.238		0–1
Muslim	.401		0–1
Other/missing	.020		0–1
Mother's age at first birth (<18 = ref.)	.223		0–1
18-19	.230		0–1
20-24	.386		0–1
25-29	.114		0–1
35+	.041		0–1
Missing	.006		0–1
Mother's marital status (Married = ref.) (*t*)	.892		0–1
Polygamous marriage	.046		0–1
Never married	.007		0–1
Divorced/separated	.020		0–1
Widowed	.013		0–1
Missing	.022		0–1
Region (Tigray = ref.)	.098		0–1
Afar	.036		0–1
Amhara	.133		0–1
Oromia	.212		0–1
Somalie	.087		0–1
Benshagul Gumuz	.030		0–1
SNNP	.286		0–1
Gambelia	.021		0–1
Harari	.043		0–1
Addis Ababa	.015		0–1
Diredwa	.039		0–1
Density (Rural = ref.)	.859		0–1
Small city	.055		0–1
Large city	.086		0–1

Note: Statistics calculated at the observation level. (*i*) varies between children; (*t*) varies over time.

Table 2 shows the main child and family characteristics by the mother's educational level in five broad categories. A large group of mothers was illiterate (934 mothers) and had zero years of education, but the largest group had some primary education (604 mothers) and there was a sizeable group with secondary education (123). There was also a small group that had (some) tertiary education (36). Note that the level of education in years varied within these groups except for the illiterate mothers. There were large social disparities by mother's education. Families with better educated mothers scored better in virtually all respects. As expected, children of more educated mothers fared better in terms of nutritional status. Children of more educated mothers were taller and heavier for their age and sex than those of less educated mothers. More educated mothers had smaller families (lower average birth order and lower average number of siblings). They were older when they had their first child and they had better educated husbands. There was no clear trend in whether the biological father was still alive or with regard to being in a monogamous marriage. Note that non-monogamous mothers were not further split-out because the groups were too small. More educated mothers were much more likely to reside in a small town or big city compared to a rural area, and the average wealth of the household increased from about 2.5 items to 7 items. Table 2 also depicts two additional general measures of food security that were not included in the analysis because they were not child-specific. More educated mothers were much less likely to report food insecurity in the past 12 months or were worried over food in the past 7 days compared to less educated mothers. Both measures indicate that children of more educated mothers in general faced better nutritional circumstances than children of less educated mothers.

**Table 2:** Descriptive statistics (unstandardized means) by mother's education (categorical) (*N* = 8275).

	Illiterate	Less than 5 years of primary education	5 years or more of primary education	(Some) secondary education	(Some) tertiary education
Child height (cm.) (*N* = 7657)	94.05	94.57	95.07	94.71	94.73
Child weight (kg.) (*N* = 8228)	13.42	13.65	13.97	14.04	14.83
Child height-for-age (z-score) (*N* = 7657)	-1.57	-1.39	-1.26	-.99	-.52
Child weight-for-age (z-score) (*N* = 8228)	-1.38	-1.22	-1.05	-.76	-.02
Child male (0–1)	.51	.51	.54	.51	.58
Child birth order (1–7+)	3.71	3.42	3.16	2.17	2.37
Child # siblings (0–7+)	3.50	3.15	2.84	1.74	1.97
					
Mother's age at first birth					
<20 years of age (0–1)	.44	.51	.51	.34	.14
<25 years of age (0–1)	.87	.91	.93	.83	.78
Mother in a monogamous marriage (0–1)	.93	.93	.92	.87	.97
Mother's education (years)	.00	2.48	6.28	10.84	15.35
Father's education (years)	1.82	3.97	5.41	10.29	13.22
Father deceased (0–1)	.09	.09	.09	.16	.05
Density					
Rural (0–1)	.94	.90	.73	.30	.10
Small (0–1)	.04	.03	.08	.20	.34
Big city (0–1)	.02	.06	.20	.50	.56
Wealth of household (# items)	2.51	3.19	4.26	5.85	7.03
Mother's report of food insecurity					
Food insecurity in past 12 months (yes)	.35	.31	.20	.08	.06
Worried over food, past 7 days (yes)	.21	.16	.15	.09	.05
					
*N* Observations	4802	1982	899	452	140
*N* Children	2532	1023	495	273	79
*N* Mothers	934	398	206	123	36

Note: all variables except height (cm.) and male are associated with mother's education at p < .001 (chi^2^ test/Spearman's rho where appropriate). Averages/proportions calculated at the observation level.

### 4.2 Regression analyses

Table 3 depicts the main results. We first turn to the first and fifth columns, which show the multi-level models for height-for-age and weight-for-age, respectively. As expected on the basis of previous research, Ethiopian boys had a better nutritional status than girls. Boys lagged less behind on the growth curve compared to girls for height-for-age (b = 0.521(0.045), p < .001) and for weight-for-age (b = 0.624(0.031), p < .001). Model 1 further shows large birth order differences both for height-for-age (b = -.715(0.061), p < .001) and for weight-for-age (b = -.447(0.041), p < .001). In line with prior studies, later born children lagged behind more than earlier born children. But note the unexpected positive effects of the number of siblings (b = 0.708(0.063), p < .001) for height-for-age (b = 0.624(0.031), p < .001), and for weight-for-age (b = 0.624(0.031), p < .001). Children in larger families were taller and heavier on average. This implies that later born children in large families may not be much worse off compared to earlier born children from small families. An auxiliary model (not shown) that does not control for the number of siblings showed a still significant negative, but much reduced, effect of birth order on height-for-age (b = -.098(0.030), p < .001) and on weight-for-age (b = -.077(0.021), p < .001). We also estimated models interacting gender*birth order, but there were only small differences by gender, so we only present the simple models. As expected by hypothesis 2, children of more educated mothers fared better in terms of nutritional status; they experienced smaller lags in height and weight (b = 0.082(0.040), p < .05) and (b = 0.096(0.028), p < .001), for height-, and weight-for-age respectively.

**Table 3:** Multi-level (labelled RE) and family fixed effect (FE) models of child and household characteristics on height-for-age and weight-for-age. Standard errors in parentheses (adjusted for multiple observations of children).

	Height-for-age				Weight-for-age			
	RE	FE	FE	FE	RE	FE	FE	FE
Model	1	2	3	4	1	2	3	4
Boy (Girl = ref.) (*i*)	.521***	.457***	.456***	.502***	.624***	.613***	.616***	.648***
	(.045)	(.049)	(.049)	(.054)	(.031)	(.033)	(.033)	(.036)
Birth order (std.) (*i*)	-.715***	-1.198***	-1.172***	-1.267***	-.447***	-.731***	-.724***	-.819***
	(.061)	(.108)	(.108)	(.108)	(.041)	(.063)	(.063)	(.065)
Number of siblings (std.) (*t*)	.708***	.235*	.226*	.280**	.434***	.124#	.123#	.162*
	(.063)	(.097)	(.097)	(.100)	(.042)	(.070)	(.070)	(.073)
Mother's education (std.)	.082*	–	–	–	.096***	–	–	–
	(.040)				(.028)			
Father's education (std.) (*i*)	.075*	.047	.046	.056	.056*	.058	.059	.058
	(.034)	(.063)	(.063)	(.069)	(.023)	(.042)	(.042)	(.049)
Wealth (std.) (*t*)	.146***	.022	.021	-.003	.130***	.043	.043	.033
	(.032)	(.052)	(.052)	(.062)	(.021)	(.032)	(.032)	(.040)
Density (Rural = ref.)								
Small city	.219	–	–	–	.199*	–	–	–
	(.135)				(.096)			
Big city	-.206#	–	–	–	.030	–	–	–
	(.125)				(.088)			
								
*Interactions withgender(Girl* = *ref.)*								
* Mother's education (std.)			.046	.085			.055	.076
			(.048)	(.070)			(.034)	(.047)
* Number of siblings (std.) (*i*) (*t*)				-.071				-.058#
				(.051)				(.034)
* Father's education (std.) (*i*)				-.006				.003
				(.063)				(.042)
* Wealth (std.) (*t*)				.029				.012
				(.062)				(.040)
* Small town (Rural = ref.)				.030				-.040
				(.253)				(.173)
* Big city (Rural = ref.)				-.492*				-.290*
*Interactions with birth order (std.)*								
* Mother's education (std.)			.159**	.246***			.042	.054
			(.056)	(.070)			(.037)	(.047)
* Number of siblings (std.) (*i*) (*t*)				.173***				.139***
				(.043)				(.029)
* Father's education (std.) (*i*)				-.064				.024
				(.047)				(.031)
* Wealth (std.) (*t*)				.058				.004
				(.041)				(.026)
* Small town (Rural = ref.)				.105				.152
				(.241)				(.170)
* Big city (Rural = ref.)				-.349				-.013
Constant	-1.818***	-1.610***	-1.583***	-1.719***	-1.403***	-1.119***	-1.114***	-1.223***
	(.126)	(.078)	(.079)	(.086)	(.088)	(.051)	(.052)	(.056)
								
*N* Observations	7657	7657	7657	7657	8228	8228	8228	8228
*N* Children	4201	4201	4201	4201	4391	4391	4391	4391
*N* Mothers	1637	1637	1637	1637	1693	1693	1693	1693

#p < .10, *p < .05, **p < .01, ***p < .001. (*i*) varies between children; (*t*) varies over time.

Note: in FE models variables that do not vary within mothers drop out (mother's education, mother's religion, mother's age at first birth, region, and density). All models include time-varying controls for mother's marital status, wave, child age, and whether the father is alive. RE model controls for mother's age at first birth, mother's religion, mother's marital status, and region are not shown.

Turning to the controls, it appears that both father's education and household wealth were associated with increased child height- and weight-for-age, and that children living in small towns did better than children in rural areas or children living in a big city (mainly Addis Ababa), especially regarding weight-for-age. The controls for whether the child's biological father was deceased and mother's age at first birth, religion, and marital status were not associated with child nutrition (not shown). Compared to a model with no predictors (model 0, not depicted), model 1 explained a substantial level of variance in height-for-age at the family and child levels; about 12% ((0.882–0.765)/.882) of the variance at the family level and about 14% ((0.280–0.217)/.280) at the child level was explained. For weight-for-age 20% at the family and 32% at the child level was explained.

Now we turn to the family fixed effects models that account for all measured and unmeasured time-constant factors that differ between families (mothers) (models 2–4) in Table 3. Variables that are constant within families are redundant and were thus omitted. The height-advantage of boys was quite similar in model 2 that only looks at within-family differences, and thus compares boys/girls with their own sisters/brothers (b = 0.457 in model 2 versus b = 0.521 in model 1). With regard to weight-for-age, there was no difference between the within and overall estimates of the boy-advantage. The birth order effect for height was on the other hand accentuated when comparing the within-family estimate to that of the overall estimate (b = -1.198 in model 2 versus b = -.715 in model 1). It is clear that higher order children had a larger height deficit than lower order children and this height deficit was larger when children were compared to their own siblings. Model 2 confirms the earlier finding that birth order was negatively associated with weight-for-age, and the negative effect of being later born was larger when comparing children with their own siblings (b = -.731 in model 2 versus b = -.447 in model 1).

Furthermore, as noted before, the height deficit seems to decrease over time in this sample, as the wave indicators were all positive (not shown). There was also a positive, albeit smaller, trend for weight-for-age. With regard to the remaining socio-economic variables that vary over time and/or between children (number of siblings, father's education, wealth, and marital status), only number of siblings and mother's marital status had a statistically significant effect. The number of children had a positive effect for both outcomes, so children in larger families were better off compared to children in smaller ones. This hints at a selection effect where families with good nutritional circumstances were more likely to have more children. It appears that children with mothers who were in a polygynous marriage were better off in terms of height-for-age, but not in terms of weight-for-age (not shown).

We now turn to model 3 which examines our hypothesis, that is whether more educated mothers compensate disadvantages associated with being a girl or a later born child on nutritional status. Model 3 introduces simultaneous interactions of mother's education with the gender and birth order of a child for height-for-age and weight-for-age. Contrary to expectations (H1), the boy advantage was not reduced when mothers were more educated. In fact, the point estimates were positive for both height and weight, although they were not significant at the p < .05 level (b = 0.046(0.048), n.s., b = 0.055(0.034), n.s.). For birth order we do see a clear buffering effect of having a more educated mother as expected by H2. The negative influence of being later born on height-for-age was reduced for children of more educated mothers as the interaction was positive and significant (b = 0.159(0.056), p < .01). The protective effect of having a more educated mother for height-for-age was about 1/7 of the original estimate (b = 0.159 for the interaction compared to -1.172 for the birth order effect). For weight-for-age we did not find a significant interaction effect (b = 0.042(0.037), n.s.).

Finally, in model 4 we included simultaneous interactions with the number of siblings to control for resource dilution effects, and with indicators of the family's and community's socio-economic position (father's education, wealth, and urbanization) to see whether the socioeconomic status of the family or the geographic area of residence are driving the observed protective effects of mother's education (Bras & Smits, 2021; Desai and Alva 1998). Most of these additional interactions with gender were not statistically significant, except the interactions of gender*large city and birth order*number of siblings. Both for height and weight, we see negative and significant interactions (b = -.492(0.206), p < .05 and -.290(0.144), p < .05). For height-for-age the boy-advantage completely disappeared in large cities (b = 0.502/-0.492) and for weight-for-age it was about halved (0.648/-0.290) compared to rural areas. Including additional interactions did not affect the interaction of gender with mother's education, which remained insignificant for both outcomes. When we look at the interactions with birth order, we see that the size of the interaction of mother's education and birth order on height-for-age (b = 0.246 versus b = 0.159) increased in model 4 compared to model 3, which may hint at suppressor effects (perhaps because more educated women live in unhealthy cities). Interestingly, the interaction of number of siblings and birth order is positive and significant for both outcomes, which implies that larger families have more resources at their disposal to partially mitigate disadvantages associated with increased birth order.

**Fig. 1:**
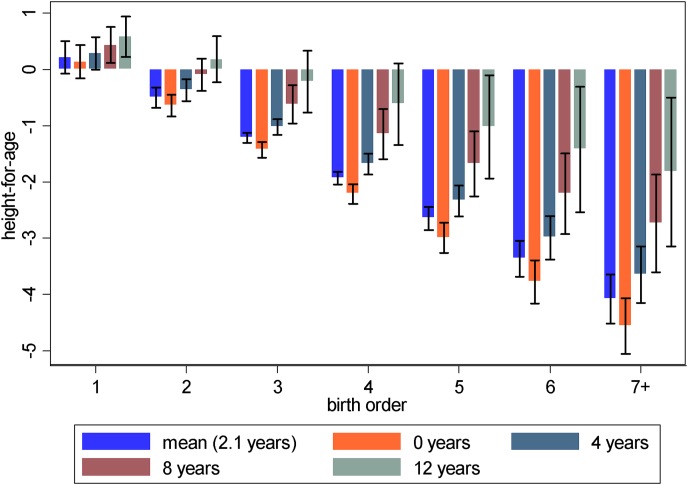
Predictive margins of height-for-age by birth order and mother's education (at the mean, 0, 4, 8 and 12 years of education) with 95% confidence intervals.

In all, we find sizeable gender and birth order differences in nutritional status. The large birth order differences were reduced for children with more educated mothers, even if we included simultaneous interactions. Especially birth order differences in height-for-age depended on mother's education, which is illustrated in Fig. 1. Note that this figure is based on a model similar to model 3 (not reported) but also controlling for interactions with the number of siblings. The figure shows that the height penalty to being a higher order child disappeared for children whose mothers have about 8 years of education or more (primary school finished/some secondary school).

### 4.3 Sensitivity analyses

We carried out two sensitivity tests to check the robustness of the results to non-linear specifications of the main variables (birth order and mother's education). First, we examined whether the results changed when we included an additional indicator for being the first born in a family and interactions for being first born with the mother's education and the other indicators. The results were unaffected by including this indicator and the interactions. Second, we used a categorical operationalization of mother's education (as in Table 2) instead of education in years and we repeated the analysis by including interactions for each of the categories. The results were again very similar and appeared quite linear. Each step higher on the educational ladder reduced the birth order differences in nutritional status. The results of these analyses are available upon request.

## 5 Conclusion and discussion

Maternal education is often seen as key to improved child health and nutrition. Little is known, however, about the redistributive potential of more educated women regarding the allocation of health and nutrition in the household. In this study we examined the association between maternal education and sibling inequalities in nutritional status. We studied height-for-age and weight-for-age of children ranging between six months and 9 years of age, who were included in three waves of a nationally representative Ethiopian household panel dataset covering the period 2011–2016.

We found clear gender inequalities between siblings in nutritional status. Both for height and weight we found a boy-advantage, with girls clearly having a poorer nutritional status. This confirms prior research and is line with patriarchal gender relations in Ethiopia and differential resource allocation practices. Maternal education did not help to straighten out boy-girl differences, thereby refuting hypothesis (H1). However, we have to keep in mind that the ESS is a very rural sample, with 85% of all households living in villages. Including an interaction between gender and living in a big city (Addis Ababa) removed the boy-advantage in height and partly in weight, likely pointing at spillover effects of an on average higher educated population, better health facilities, and more equal gender norms.

We found a steep birth order gradients for height and weight. The magnitude of these birth order differences is large and, with regard to height, comparable to what has been found for India (Jayachandran & Pande, 2017). Possible causes include patrilineal and patrilocal property ownership and inheritance practices. Moreover, first-borns, particularly sons, enter the labor market first and remit their earnings to the household budget. Furthermore, cultural norms regarding the specific sibling that has to provide parental old-age support may also play a role. Strikingly, as expected (H2), more educated mothers buffered birth order disparities in nutritional status. The benefit of having a more educated mother only plays out in long-run nutritional status, given the attenuated height differences, and not so much in weight. Additional controls showed that neither the socioeconomic status of the family nor the geographic area of residence were driving the observed protective effect of mother's education on height.

Our study has a number of limitations. First, the precise nature of the attrition and panel follow-up of the sample is unclear. Given this uncertainty, we cannot precisely locate potential biases caused by the non-inclusion of migratory and hard-to-find groups. Second, the sample has a skewed distribution concerning maternal education. There are many women with no education, and only a few with higher, tertiary education. However, we carefully checked for possible distortive consequences, and found that the unbalance in education does not influence our results. Third, although the sample was based on nationally representative data care should be taken in generalizing the results. Fourth, we were not able to investigate the effect of birth order in combination with older siblings’ gender to better understand how these may jointly influence child health inputs and nutrition. Fifth, our study could not explicitly identify the precise mechanisms underlying the link between maternal education and child health: knowledge, income, or negotiating power.

Future studies could explore birth order in combination with the gender of older siblings to better understand differences in nutritional status stemming from early nutritional inputs, as well as short-versus long-term consequences of undernutrition at critical periods. To better understand the role of bargaining power and other mechanisms in the link between maternal education and child nutrition, further research may compare single, married, and remarried mothers, biological and stepchildren, as well as capitalize on variation in maternal education within polygynous households. More in-depth studies on regional, ethnic, and religious differences in child preferences and child nutrition in Ethiopia are also desirable. Finally, qualitative research is needed to better understand the mechanisms underlying the protective effects of maternal education.

In all, our findings make a significant contribution to the literature on maternal education and child nutrition in sub-Saharan Africa by adding a within-family component. Recent research shows that while global health is improving, health inequalities are on the rise (Deaton, 2013). Our study demonstrates the importance of sibling inequalities, which may be long-lasting and influence later life outcomes, such as education, income, and health. Knowledge of sibling disparities may help donors, governments, and international development agencies to improve access to children in the most disadvantageous positions in the household. Moreover, the beneficial impact of maternal education, counteracting some of these within-family inequalities, asks for continued investments in girls' and women's education (Marphatia et al., 2016).

## Financial disclosure statement

The study was conducted without external funding

## Author statement

Hilde Bras: conceptualization; writing.

Jornt Mandemakers: methodology; formal analysis.

## Ethical statement

Ethical clearance for the Ethiopia Socioeconomic Survey was obtained by the Central Statistical Agency of Ethiopia (CSA) and the World Bank.

## Declaration of competing interest

The authors declare that they have no competing interests.
